# Gd(III)-DOTA-modified sonosensitive liposomes for ultrasound-triggered release and MR imaging

**DOI:** 10.1186/1556-276X-7-462

**Published:** 2012-08-17

**Authors:** Suk Hyun Jung, Kyunga Na, Seul A Lee, Sun Hang Cho, Hasoo Seong, Byung Cheol Shin

**Affiliations:** 1 Research Center for Medicinal Chemistry, Division of Drug Discovery Research, Korea Research Institute of Chemical Technology, 141 Gajeong-ro, Yuseong-gu, Deajeon, 305-600, South Korea

**Keywords:** Liposome, Ultrasound sensitivity, Contrast agent, Intracellular uptake, Doxorubicin

## Abstract

Ultrasound-sensitive (sonosensitive) liposomes for tumor targeting have been studied in order to increase the antitumor efficacy of drugs and decrease the associated severe side effects. Liposomal contrast agents having Gd(III) are known as a nano-contrast agent system for the efficient and selective delivery of contrast agents into pathological sites. The objective of this study was to prepare Gd(III)-DOTA-modified sonosensitive liposomes (GdSL), which could deliver a model drug, doxorubicin (DOX), to a specific site and, at the same time, be capable of magnetic resonance (MR) imaging. The GdSL was prepared using synthesized Gd(III)-DOTA-1,2-distearoyl-*sn*-glycero-3-phosphoethanolamine lipid. Sonosensitivity of GdSL to 20-kHz ultrasound induced 33% to 40% of DOX release. The relaxivities (*r*_1_) of GdSL were 6.6 to 7.8 mM^−1^ s^−1^, which were higher than that of MR-bester®. Intracellular uptake properties of GdSL were evaluated according to the intensity of ultrasound. Intracellular uptake of DOX for ultrasound-triggered GdSL was higher than that for non-ultrasound-triggered GdSL. The results of our study suggest that the paramagnetic and sonosensitive liposomes, GdSL, may provide a versatile platform for molecular imaging and targeted drug delivery.

## Background

Liposomes are spherical vesicles composed of phospholipid bilayer membranes. In the field of targeted drug delivery, liposomes have been extensively studied in an attempt to enhance the therapeutic efficacy of various drugs [1,2]. Many studies have reported that modification of the surface of liposomes with a hydrophilic moiety such as polyethylene glycol (PEG) can increase the circulation time of the liposomes in the bloodstream due to reduced uptake of the liposomes by the reticuloendothelial system (RES) [3-7]. However, utilization of liposomes as drug-carrying vehicles for intracellular delivery of anticancer drugs loaded in the liposomes is limited due to the lack of specific interaction between liposomal carriers and the target cells [8]. Therefore, to overcome this problem, targeted drug delivery systems such as thermo-, pH-, ultrasound-, and optical-sensitive liposomes have been studied [9,10]. In particular, ultrasound-sensitive (sonosensitive) liposomes for controlled drug release at the target site have been studied in order to increase the antitumor efficacy of drugs and decrease the associated side effects [11,12].

Magnetic resonance (MR) is widely used in diagnostic medicine to image pathological areas. Usually, accumulation of contrast agents is essential to achieve successful MR imaging (MRI) and high-resolution images [13,14]. Most MRI contrast agents are based on either iron oxide particle or gadolinium (III) (Gd(III))-chelated complexes. Gd(III)-based contrast agents have a low *r*_2_/*r*_1_ ratio and are frequently used to generate positive contrast (increased signal intensity) in *T*_1_-weighted images. Recently, various nanoscale carriers such as liposomes, micelles, and polymeric nanoparticles have been modified or incorporated with the MRI contrast agent Gd(III) [14,15]. Liposomal nanocarriers are able to carry multiple reporter moieties such as peptides and antibodies for the efficient and selective delivery of contrast agents into the pathological sites [16].

The objective of this study was to develop a novel liposomal carrier that could provide a convenient ultrasonic therapy, such as high- or low-intensity focused ultrasound therapy, by MR image guidance and, moreover, a possibility of ultrasound-mediated targeted drug delivery during ultrasonic therapy. In the current study, we prepared Gd(III)-DOTA-modified sonosensitive liposomes (GdSL), which could deliver doxorubicin (DOX) to a specific site and, at the same time, enhance signal intensity in regions of accumulation on *T*_1_-weighted MRI. The GdSL was prepared using synthesized Gd(III)-DOTA-1,2-distearoyl-*sn*-glycero-3-phosphoethanolamine (DSPE) lipid. Sonosensitivity and MR properties of the GdSLs with varying lipid ratios were investigated. Furthermore, intracellular uptake property of the GdSL was evaluated according to the intensity of ultrasound.

## Methods

### Materials

1,2-Distearoyl-*sn*-glycero-3-phosphoethanolamine, 1,2-distearoyl-*sn*-glycero-3-phosphoethanolamine-*N*-[methoxy(polyethylene glycol)-2000] (DSPE-mPEG2000), and cholesterol (CHOL) were purchased from Avanti Polar Lipids Inc. (Alabaster, AL, USA). Doxil® was purchased from ALZA Corporation (Mountain View, CA, USA). Doxorubicin hydrochloride, *N,N′*-diisopropylethylamine (DiPEA), *N,N,N′,N*′-tetramethyl-O-(*N*-succinimidyl) uranium hexafluorophosphate (HSTU), trifluoroacetic acid (TFA), and gadolinium (III) acetate hydrate (Gd(III) (OAc)_3_) were purchased from Sigma-Aldrich Chemical Co (St. Louis, MO, USA). Fetal bovine serum (FBS), penicillin-streptomycin, paraformaldehyde, and Dulbecco's modified Eagle medium (DMEM) were purchased from Gibco BRL/Life Technologies (New York, NY, USA). Tri-tert-butyl 1,4,7,10-tetraazacyclododecane-1,4,7,10-tetraacetate (protected DOTA) was purchased from Tokyo Chemical Industry Corporation (TCI, Tokyo, Japan). All other materials were of analytical grade and used without further purification.

### Synthesis of Gd(III)-DOTA-DSPE

**Figure 1:**
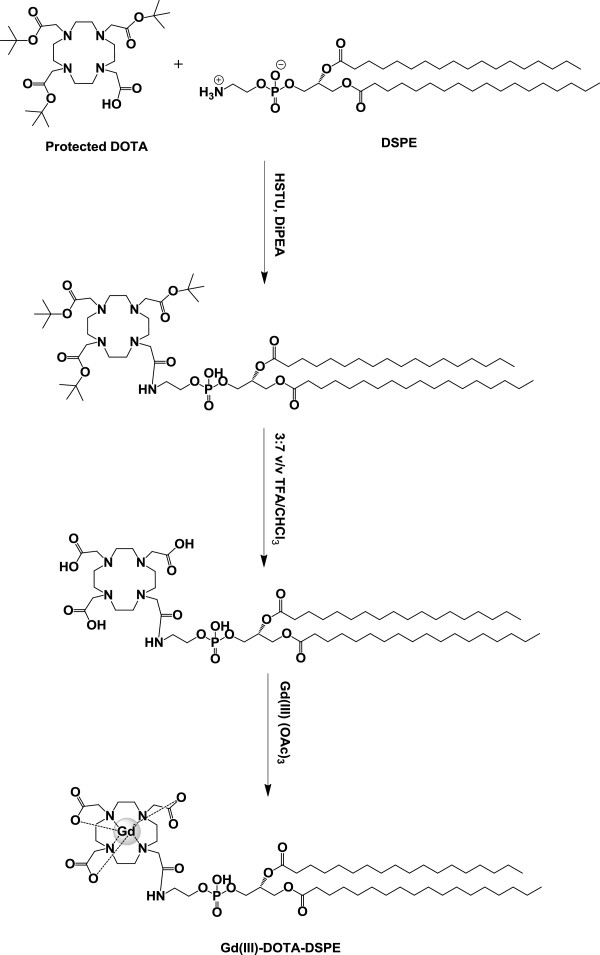
Synthesis of Gd(III)-DOTA-DSPE for the preparation of GdSLs.

Gd(III)-DOTA-DSPE was synthesized as shown in Figure 1[17-19]. Protected DOTA (0.9 mmol) was added to 5 ml of dry dimethylformamide containing 0.9 mmol of HSTU and 3.2 mmol of DiPEA under a nitrogen atmosphere, and the mixture was stirred for 1 h at room temperature. The obtained solution was added to 10 ml of chloroform containing 0.8 mmol of DSPE, stirred for 3 h at 65°C, and incubated overnight at room temperature. After evaporation of the solution with toluene (2 × 20 ml) and chloroform (2 × 20 ml) using a rotary evaporator (Buchi Rotavapor R-200, Flawil, Switzerland), the product was precipitated in water/methanol (1:1 *v*/*v*), and the precipitate was isolated by centrifugation. The resulting pellet was dissolved in chloroform, and the solution was concentrated under vacuum. The resulting solid was redissolved in 20 ml of diethyl ether and washed with water (2 × 10 ml). The organic phase was collected and concentrated in vacuum to obtain powder. The prepared powder was dissolved in 10 ml of TFA/chloroform (3:7, *v*/*v*) and stirred for 17 h at room temperature. The solution was evaporated with toluene (2 × 20 ml) and chloroform (2 × 20 ml) using a rotary evaporator, precipitated in acetonitrile, and dried under vacuum. A solution of the yellowish solid in 9 ml of chloroform was mixed with a solution of 0.42 mmol Gd(III) (OAc)_3_ in 5 ml of methanol/water (10:1, *v*/*v*), adjusted to pH 6.5 with pyridine, and stirred overnight at room temperature. The solution was concentrated under reduced pressure and co-evaporated with methanol/toluene (1:1, *v*/*v*, 2 × 10 ml) and chloroform (2 × 10 ml), resulting in Gd(III)-DOTA-DSPE as a yellowish solid. The yield of Gd(III)-chelated Gd(III)-DOTA-DSPE was 80%. Gd(III)-DOTA-DSPE, ^1^ H-NMR (CDCl_3_): δ = 7.73 (s, 1 H, *NH*), 4.24 (m, 1 H, *CH*), 2.34 (m, 4 H, *CH*_*3*_*(CH*_*2*_*)*_*15*_*CH*_*2*_*CO*), 1.27 (m, 60 H), 0.91 (t, 6 H, *(CH*_*2*_*)*_*15*_*CH*_*3*_), and MALDI-TOF (negative mode): m/z [M-H^−^, calculated = 1,287.1 Da., observed = 1,287.2 Da.

### Preparation of Gd(III)-DOTA-modified sonosensitive liposomes

Gd(III)-DOTA-modified sonosensitive liposomes (GdSL) and sonosensitive liposomes (SL) without Gd(III)-DOTA-DSPE were prepared by thin-film hydration and sequential extrusion method. The loading of DOX into the aqueous core of the liposomes was carried out using the remote loading method using an ammonium sulfate transmembrane gradient [20,21]. The lipid compositions and molar ratios of the lipids for the preparation of the liposomes were as follows: (1) GdSL1, Gd(III)-DOTA-DSPE/CHOL/DSPE-mPEG = 31:15:4; (2) GdSL2, Gd(III)-DOTA-DSPE/CHOL/DSPE-mPEG/DSPE = 20:15:4:11; (3) GdSL3, Gd(III)-DOTA-DSPE/CHOL/DSPE-mPEG/DSPE = 10:15:4:21; and (4) SL, CHOL/DSPE-mPEG/DSPE = 15:4:31. The lipids for each liposome formulation were dissolved in 3 ml of chloroform to give 16.67 mM of total lipid concentration (GdSL1, 18.97 mg/ml; GdSL2, 16.97 mg/ml; GdSL3, 15.17 mg/ml; and SL, 13.41 mg/ml) and dried to a thin film using a rotary evaporator. The film was hydrated with 3 ml of 300 mM ammonium sulfate solution, and the liposome suspension was extruded sequentially five times through polycarbonate membrane filters (Whatman, Piscataway, NJ, USA) with a pore size of 200 and 100 nm using a high-pressure extruder (Northern Lipids Inc., Burnaby, Canada). Unloaded ammonium sulfate was removed by dialysis in distilled water for 24 h at 4°C using a cellulose dialysis tube (MWCO, 12,000 to 14,000; Viskase Co., Darien, IL, USA). DOX solution (2 mg/ml) was added to the liposomal solution (1:1, *v*/*v*) and incubated for 2 h at 75°C. The mixture was dialyzed for 48 h at 4°C to remove the unloaded DOX. The DOX-loaded liposomes were stored at 4°C until use.

The concentration of DOX in the liposomes was measured using a UV-vis spectrophotometer at 497 nm (UV-mini; Shimadzu, Tokyo, Japan), and the encapsulation efficiency was calculated according to the following equation:

(1) $$\text{Encapsulation efficiency}\;\left(\%\right)=\frac{F_{t}}{F_{i}}\times 100$$

where *F*_t_ is the concentration of DOX in the liposomes after the dissolution of DOX-loaded liposomes in an organic solvent mixture consisting of chloroform/methanol (2:1, *v*/*v*), and *F*_i_ is the initial concentration of DOX. The particle size and zeta potential of the liposomes were measured using an electrophoretic light scattering spectrophotometer (ELS-Z, Otuska Electronics Co., Tokyo, Japan). The amount of chelated Gd(III) was measured using an inductively coupled plasma-atomic emission spectrometer (Ultima-C, Jobin Yvon, Longjumeau, France).

### Ultrasound- and temperature-triggered drug release from liposomes

Ultrasound-triggered release of DOX from GdSL and SL was conducted using a 20-kHz ultrasound system (VC 750; Sonic and Materials, Inc, Newtown, CT, USA). The intensity of the generated ultrasound was determined using a calorimetric method [22,23]. For input power levels of 80, 160, and 240 W, the calculated intensity levels were 14.8, 27.8, and 63.5 W/cm^2^, respectively. The liposomal solutions were diluted in a ratio of 1:4 (*v*/*v*) with PBS (pH 7.4) and exposed to a continuous mode (100% duty cycle) of ultrasound for 1 min at intensity levels of 14.8, 27.8, and 63.5 W/cm^2^, respectively. During the ultrasound irradiation, the temperature of each sample was controlled to be below 50°C. Temperature-mediated release from GdSL was evaluated using MULTI-BLOK (Lab-Line Instruments, Melrose Park, IL, USA). The liposomal solutions were exposed to 37°C or 50°C for 1 min.

The release of DOX from liposomes was measured by fluorescence spectrophotometry. The excitation and emission wavelengths were 487 and 595 nm, respectively. The percentage of DOX release from the liposomes was calculated as follows:

(2) $$\text{Drug release}\;\left(\%\right)=\frac{\left(F_{t}-F_{0}\right)}{\left(F_{\max}-F_{0}\right)}\times 100$$

where *F*_t_ is the fluorescence intensity of the liposome sample after a given duration (t) of ultrasound irradiation, *F*_0_ is the initial background fluorescence of the liposome sample prior to ultrasound irradiation, and *F*_max_ is the fluorescence intensity of DOX in the liposomes after dissolution of DOX-loaded liposomes in an organic solvent mixture consisting of chloroform/methanol (2:1, *v*/*v*) [24]. The release test was performed on three independent samples of each liposome.

### Morphology of Gd(III)-DOTA-modified sonosensitive liposomes

The morphology of GdSL was observed by cryogenic transmission electron microscopy (cryo-TEM; Tecnai G2 Spirit, FEI Company, Hillsboro, OR, USA). Samples for the cryo-TEM observation were prepared using a controlled-environment vitrification system. Five microliters of the sample were put on a carbon film supported by a copper grid and blotted with filter papers to obtain a thin liquid film on the grid. The sample-loaded grid was quenched in liquid ethane at −180°C and transferred to liquid nitrogen. The acceleration voltage was 80 kV, and the working temperature was −180°C. The images were recorded with a CCD camera (Proscan GmbH, Scheuring, Germany) and an analysis software (Soft Imaging System, GmbH, Munster, Germany) [25].

### Relaxivity measurement

The liposomal samples were prepared in the range of 0.05 to 0.40 mM of Gd(III) concentration. The longitudinal relaxation time (*T*_1_) of each sample was measured by saturation recovery method using a 4.7-T MR system (Bruker-biospin, Ettlingen, Germany). Relaxivity (*r*_1_, in units of mM^−1^ s^−1^) was obtained from the slope of the linear fit of the inverse of *T*_1_ as a function of Gd(III) concentration. *T*_1_-weighted MR images were obtained using a heavily *T*_1_-weighted fast spoiled gradient echo sequence. Scans were performed with the following imaging parameters: repetition time (TR) = 8.0, 6.0, 4.0, 2.5, 0.5, 0.2, and 0.07 s; echo time (TE) = 7.8 ms; flip angle (FA) = 180°; field of view (FOV) = 40 × 50 mm^2^; image matrix = 128 × 128 mm^2^; and number of signal average = 5.

### Intracellular uptake of DOX from ultrasound-triggered liposomes

For the experiments on intracellular uptake of DOX from liposomes, B16F10 murine melanoma cells were cultured in DMEM supplemented with 10% (*v*/*v*) heat-inactivated FBS and 10 μl/ml penicillin-streptomycin. The cultures were sustained at 37°C in a humidified incubator containing 5% CO_2_. The cells were maintained within their exponential growth phase. The intracellular uptake of DOX from liposomes was determined by flow cytometry analysis [24,26]. B16F10 cells were transferred to 24-well tissue culture plates at a density of 1 × 10^5^ cells/well and incubated for 12 h at 37°C. The liposomal DOX solutions were diluted in a ratio of 1:4 (*v*/*v*) with PBS (pH 7.4) just prior to the experiments. The diluted liposomal solutions were irradiated by ultrasound using a 20-kHz ultrasound transducer in a continuous mode (100% duty cycle) at an intensity level of 14.8, 27.8, or 63.5 W/cm^2^ for 2 min at 37°C. The culture medium was replaced with the ultrasonically irradiated liposomal DOX solution diluted in culture media at a concentration of 15 μg of DOX/ml and then incubated for 45 min. The culture medium was then removed, and each well was washed with PBS (pH 7.4). To fix the cells, 300 μl of paraformaldehyde (5%, *v*/*v*) was added to each well. The fluorescence intensities of the sample were determined by flow cytometry with a FACScan (Becton Dickinson, San Jose, CA, USA). Cell-associated DOX was excited with an argon laser (488 nm), and fluorescence was detected at 560 nm. Data of 10,000 gated events were collected and analyzed with the CELL Quest software.

## Results and discussion

### Physical properties of liposomes

The physical properties of various GdSLs and SL were evaluated by measuring their mean particle diameter, zeta potential, DOX-loading efficiency, and amount of Gd(III), as summarized in Table 1. The mean particle diameter of SL was approximately 129 nm, and the particle diameter of GdSL was increased according to the amount of Gd(III)-DOTA-DSPE in the lipid bilayer of the liposome. The zeta potential value of the liposomes was approximately −20 to −30 mV due to the PEG and the Gd(III)-DOTA complex having lone pairs of oxygen atom electrons. DSPE-mPEG2000 incorporated in pegylated liposomes is known to inhibit opsonization and RES uptake of the liposomes by forming hydrodynamic layer on them and hence prolong circulation time of the encapsulated drugs in the bloodstream [27]. CHOL in liposome plays a role in increasing the stability of the liposome. It was reported that incorporation of CHOL to drug-carrying vehicles such as liposomes could increase their stability in the bloodstream [28]. The amount of Gd(III) in GdSLs was proportional to the increase of Gd(III)-DOTA-DSPE content in the lipid composition. The Gd(III)-DOTA-DSPE was used as a lipid having magnetic resonance effect for contrast agents. Recently, it was reported that DSPE as the main lipid component could enhance sonosensitivy of the liposomes [29]. Therefore, DSPE was herein used as a sonosensitive lipid. The DOX-loading efficiency of GdSLs was lower by approximately 35% to 56% than that of SL. The Gd(III)-DOTA complex may induce steric effect and mechanical stress in the membrane of lipid bilayers due to the repulsive forces between the Gd(III)-DOTA complex and PEG [11,30].

**Table 1:** Physical properties of liposomes

**Liposome formulation**	**Mean particle diameter**	**Zeta potential**	**DOX-Encapsulation efficiency**	**Amount of Gd(III)**
**(molar ratio of lipids)**				
	**(nm)**	**(mV)**	**(%)**	**(mM)**
GdSL1	157.1 ± 8.9	−27.6 ± 4.2	46.6 ± 12.7	4.60 ± 0.16
Gd(III)-DOTA-DSPE/CHOL/DSPE-mPEG2000 = 31:15:4)				
GdSL2	156.4 ± 5.1	−21.9 ± 1.0	41.7 ± 9.7	3.17 ± 0.01
Gd(III)-DOTA-DSPE/CHOL/DSPE-mPEG2000/DSPE = 20:15:4:11)				
GdSL3	131.4 ± 9.1	−31.6 ± 7.2	62.9 ± 1.5	2.01 ± 0.31
Gd(III)-DOTA-DSPE/CHOL/DSPE-mPEG2000/DSPE = 10:15:4:21)				
SL	129.1 ± 6.6	−31.9 ± 2.3	97.5 ± 0.4	ND
(CHOL/DSPE-mPEG2000/DSPE = 15:4:31)				

ND, not detected (amount of Gd(III) could not be measured because there is no Gd(III)).

### Ultrasound- and temperature-triggered drug release from liposomes

**Figure 2:**
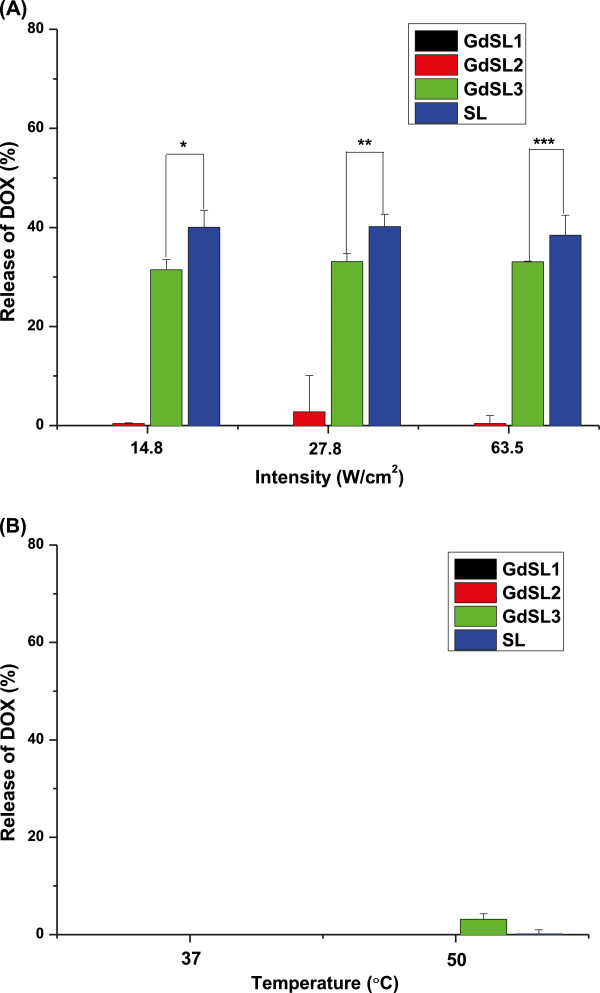
**Release pattern of DOX from GdSLs and SL.** (**A**) The release of DOX from the liposomes after irradiation of 20-kHz ultrasound at various intensities and (**B**) the release of DOX from the liposomes after incubation at 37°C or 50°C. Molar ratio of Gd(III)-DOTA-DSPE, CHOL, and DSPE-mPEG2000 for GdSL1 was 31:15:4. Molar ratios of Gd(III)-DOTA-DSPE, CHOL, DSPE-mPEG2000, and DSPE for GdSL2 and GdSL3 were 20:15:4:11 and 10:15:4:21, respectively. Molar ratio of CHOL, DSPE-mPEG2000, and DSPE for SL was 15:4:31. All samples were treated for 1 min. Mean and S.D. are shown (*n* = 3) (asterisk, two asterisks, and three asterisks denote *p* < 0.05, Student's *t* test).

The release profile of DOX from GdSL and SL was investigated under various intensities (14.8, 27.8, and 63.5 W/cm^2^) of 20-kHz ultrasound and temperatures (37°C and 50°C), as shown in Figure 2. The ultrasound-triggered release of DOX from the liposomes was proportional to the increase of DSPE mole ratio in the lipid composition (Figure 2A). GdSL3 and SL showed a high DOX release of 33% to 40% by ultrasound irradiation. Temperature-dependent release of DOX from GdSL3 was not measured at 37°C and showed a release of approximately 3.2% at 50°C (Figure 2B). The results indicate that the drug release from the sonosensitive liposomes, GdSL and SL, could not be triggered at normal body temperature but could be triggered mainly by cavitation.

**Figure 3:**
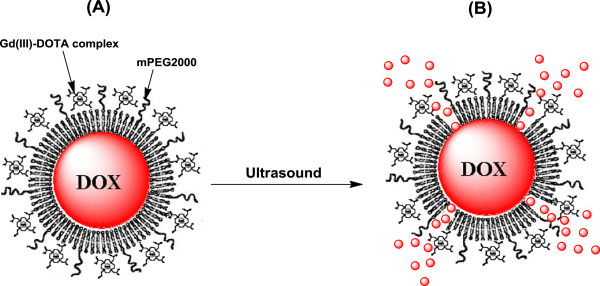
**Schematic representation of ultrasonically triggered GdSL.** (**A**) DOX-loaded GdSL and (**B**) ultrasound-triggered release of DOX from GdSL.

Recently, Evjen et al. reported that sonosensitivity of the liposomes is related to the ability of DSPE to form inverted hexagonal structures under high temperature or pressure [29]. Ultrasound irradiation-induced cavitations can generate high pressure or temperature in the liposome membrane [10,11]. Phase transitions of liposomes have been known to be induced by pressure and/or temperature changes. DSPE in the liposomal bilayer undergoes a thermotropic phase transition from the lamellar liquid-crystalline to the inverted hexagonal phase by cavitation because the long fatty acids occupy larger volume than the polar head groups [29,31]. The phase transition might induce local defects or polymorphic phase transitions within micro-rafts or the whole liposome bilayer during ultrasound irradiation, further leading to drug release by membrane rupture, as shown in Figure 3.

### Morphology of ultrasound-irradiated liposomes

**Figure 4:**
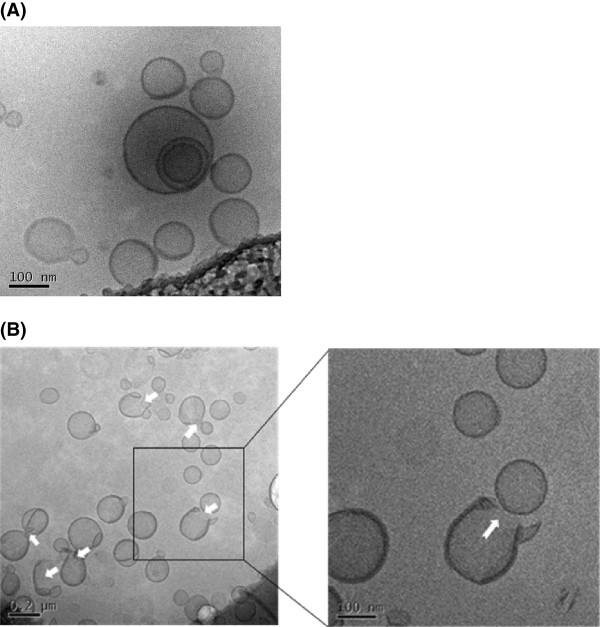
**Cryo-TEM images of (A) GdSL3 and (B) ultrasound-irradiated GdSL3.** GdSL3 was irradiated by 20-kHz ultrasound for 5 min at an intensity of 63.5 W/cm^2^. The arrows indicate the snapped liposomal membrane.

The morphology of GdSL3 observed by cryo-TEM is shown in Figure 4. The majority of non-ultrasound-irradiated GdSL3 was observed as a unilamellar liposome structure having approximately 100 to 150 nm of particle (Figure 4A). The mean particle diameter of GdSL3 observed by cryo-TEM was similar to the value of 131.4 ± 9.1 nm analyzed by light scattering method using a particle size analyzer (see Table 1). However, ultrasound-irradiated GdSL3 was observed in the shape of snapped liposomal membrane (>20%), as shown in Figure 4B. The results indicate that ultrasound irradiation could induce rupture of the liposomal membrane by local phase transition, further leading to drug release, as shown in Figure 3.

### Magnetic resonance property of liposomes

MRI is one of the most powerful techniques currently used in medical diagnostics such as tumor detection and vascular imaging. Gd-based complexes, such as Gd(III)-DOTA and Gd(III)-DTPA using paramagnetic material, are known as the most effective *T*_1_ agents [16]. The MR images of contrast agents are based on the same principles of nuclear magnetic resonance (NMR). The MR image of contrast agents is related to the relaxation behavior of hydrogen nuclei of water. The principle mechanism for Gd(III)-complexes is due to the interaction of an inner-sphere water molecule with the paramagnetic Gd(III) ion having ninth coordination site, leading to the subsequent magnetic relaxation of the water molecule [32]. The signal intensity of the image is related to the longitudinal relaxation time (*T*_1_), and a shortened *T*_1_ provides improved images [33].

**Figure 5:**
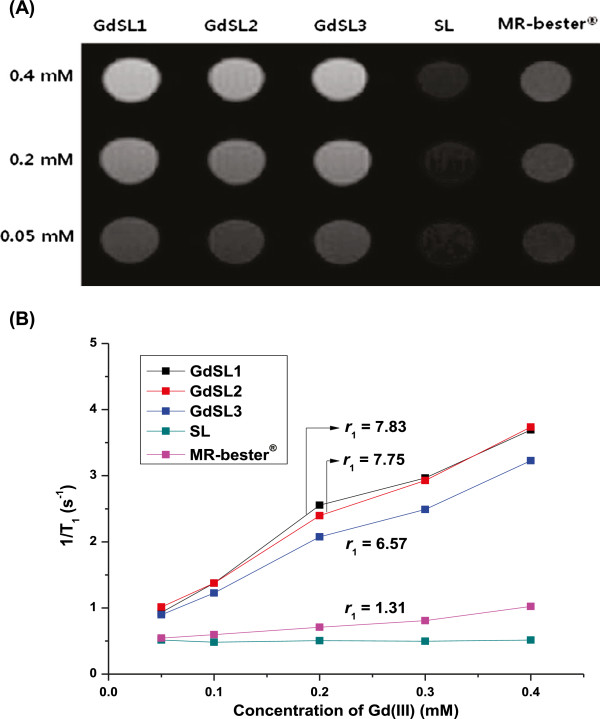
**Magnetic resonance properties of GdSLs.** (**A**) *T*_1_-weighted MR images of GdSLs and MR-bester® at different concentrations of Gd(III). The MR images of SL were taken together for comparison. (**B**) Relaxivities (*r*_1_, in units of mM^−1^ s^−1^) of GdSLs and MR-bester®. Relaxivity was obtained from the slope of the linear fit of the inverse of the measured *T*_1_ (longitudinal relaxation time) as a function of Gd(III) concentration. 1/*T*_1_ data of SL without Gd(III)-DOTA-DSPE were presented as negative controls.

Figure 5A shows *T*_1_-weighted MR images of GdSLs and a commercial contrast agent, MR-bester® (Taejoon Pharmaceuticals Co., Ltd., Seoul, Korea), at different concentrations of Gd(III) (0.4, 0.2, and 0.05 mM). The MR images of various GdSLs showed similar brightness and looked brighter compared to MR-bester® at the same concentration of Gd(III). The brightness of MR imaging of GdSL was proportional to the increase in Gd(III) concentration. The MR images of SL were very dark because they did not contain Gd(III). The relaxivity (*r*_1_) values of GdSLs were 6.57 to 7.83 mM^−1^ s^−1^, which was approximately 5.0 to 6.0 times higher than that of MR-bester®, as shown in Figure 4B. These results indicate that GdSLs could induce strong relaxivity compared to MR-bester®. Generally, Gd(III) complexes on the liposomal surface are known to improve ionic relaxivity compared to Gd(III)-entrapped liposomes [16]. The NMR dispersion profiles of liposomal contrast agents show a typical peak at higher frequencies [14,15]. This is in agreement with the increase in the rotational correlation times when compared with low molecular weight Gd(III) complexes. Based on these results, GdSL3 was selected as an optimized carrier for the cellular uptake study because it showed high sonosensitivity and relaxivity.

### Intracellular uptake of DOX released from ultrasound-triggered liposomes

**Figure 6:**
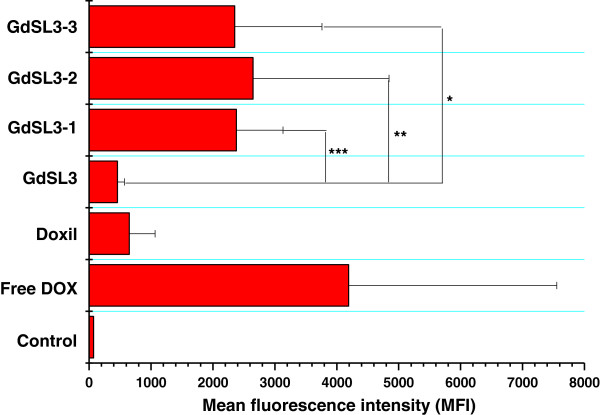
**Intracellular uptake of DOX released from ultrasound-triggered GdSL3.** MFI was determined by flow cytometry analysis. GdSL3-1, GdSL3-2, and GdSL3-3 were irradiated by 20-kHz ultrasound for 2 min at intensity levels of 14.8, 27.8, and 63.5 W/cm^2^, respectively. GdSL3, free DOX, and Doxil® were not irradiated with ultrasound. Mean and S.D. are shown (*n* = 3) (asterisk, two asterisks, and three asterisks denote *p* < 0.1, Student's *t* test).

To investigate the intracellular uptake of DOX released from ultrasound-triggered liposomes, the amount of accumulated DOX in B16F10 cells was measured by flow cytometry. The results are shown in Figure 6. Intracellular uptake of DOX released from ultrasound-irradiated GdSL3 was higher than that from GdSL3 or Doxil®. The mean fluorescence intensity (MFI) values for ultrasound-irradiated GdSL3 were approximately 3.7 to 5.4-fold higher than that for GdSL3. The results indicate that the triggered DOX release by ultrasound irradiation could increase intracellular uptake of DOX compared to that of liposomal DOX. Free DOX is known to enter cells by diffusion, leading to high cellular uptake compared to the liposomal DOX, and the liposomes modified with a DOX-phospholipid conjugate can increase the cellular uptake of DOX compared to the unmodified ones [24,26]. GdSL3 and Doxil® exhibited low intracellular uptake of DOX because GdSL3 and Doxil® with the anionic surface charges could have electrostatic repulsion with the cellular membrane. Ultrasound-irradiated GdSL3 showed greater intracellular uptake of DOX because it induced the burst release of DOX by cavitation in 1 min, as shown in Figure 2. Additionally, the intracellular uptake of DOX according to the intensity of ultrasound showed a similar increase in intracellular uptake.

## Conclusions

Dual functional Gd(III)-DOTA-modified sonosensitive liposomes were prepared and evaluated for their sonosensitivity, MR properties, and *in vitro* intracellular uptake. GdSL showed excellent contrast efficiency compared to a commercial contrast agent, MR-bester®, and increased intracellular uptake due to the ultrasound-triggered release of the drug. Therefore, GdSL could deliver drugs to specific sites by ultrasound irradiation and, at the same time, allow MR imaging due to enhanced *T*_1_ relaxivity. The results of our study suggest that the novel liposomal carrier may provide a convenient ultrasonic therapy by MR image guidance and, moreover, a possibility of ultrasound-mediated targeted drug delivery during ultrasonic therapy.

## Competing interests

The authors declare that they have no competing interests.

## Authors' contributions

SHJ performed the preparation and characterization of the liposomes, participated in the studies on their ultrasound-mediated drug release, magnetic resonance properties, and *in vitro* cellular uptake, analyzed the data, and drafted the manuscript. KN participated in the studies on the magnetic resonance properties and the cellular uptake. SAL participated in the preparation and characterization of the liposomes. SHC participated in design of the study, interpretation of the data, and discussion on the results. HS participated in analysis and interpretation of the data and revision of the manuscript. BCS conceived of the study, designed the study and experiments, interpreted the data, discussed the results, helped to draft and revise the manuscript, and approved the manuscript. All authors read and approved the manuscript.
